# Association of the Severity of Hypertensive Disorders in Pregnancy with Birthweight, Childhood Obesity, and Blood Pressure at Age 7

**DOI:** 10.3390/nu15143104

**Published:** 2023-07-11

**Authors:** Yan Chen, Yiwen Wang, Yanjun Li, Guodong Ding, Yongjun Zhang

**Affiliations:** 1 Department of Pediatrics, Xinhua Hospital, Shanghai Jiao Tong University School of Medicine, Shanghai 200092, China; chenyan783563@163.com (Y.C.); wangyiwenwin@163.com (Y.W.); 18545529468@163.com (Y.L.); 2 Ministry of Education-Shanghai Key Laboratory of Children’s Environmental Health, Xinhua Hospital, Shanghai Jiao Tong University School of Medicine, Shanghai 200092, China

**Keywords:** hypertensive disorders in pregnancy, macrosomia, body mass index, blood pressure, placental pathology

## Abstract

We aimed to examine the effects of various severities of hypertensive disorders in pregnancy on birthweight, blood pressure (BP), and body mass index in offspring at age 7. In the China Labor and Delivery Survey and the United States Collaborative Perinatal Project (CPP), the relationship of the severity of hypertensive disorders and nutritional and cardiovascular outcomes in offspring was assessed using a multivariable logistic and general linear regression model. In both datasets, those with gestational hypertension were more likely to deliver large for gestational age (LGA) and macrosomia (adjusted odds ratios (aOR) ranged from 1.29 to 1.91), as well as low birth weight (LBW) neonates (aOR ranged from 1.23 to 3.56), compared with normotensive mothers. In the CPP, when gestational hypertension was further stratified into mild and severe, only those with mild gestational hypertension (the mild group) were more likely to deliver macrosomia and LGA (aOR ranged from 1.25 to 1.32). Others (severe gestational hypertension and preeclampsia/eclampsia) were closely related to LBW and small for gestational age (aOR ranged from 1.27 to 2.77). Moreover, children of mothers in the mild group tended to be overweight/obese and had elevated diastolic BP. We concluded that the severity of hypertensive disorders had different effects on birthweight, childhood overweight, and BP.

## 1. Introduction

Hypertensive disorders in pregnancy, consisting of preeclampsia and gestational hypertension, are major causes of neonatal, fetal, and maternal mortality and morbidity and adverse health outcomes for offspring in later life [1,2,3,4].

Maternal preeclampsia/eclampsia and gestational hypertension have been related to offspring birth size; however, their effects are inconsistent. Most often, the offspring are smaller than expected. Bakker et al. found that hypertensive disorders in pregnancy are related to small-for-gestational-age infants [5]. In contrast, Xiong et al. concluded a significant relationship between gestational hypertension, preeclampsia, and large-for-gestational-age infants [6]. The potential mechanism underlying these opposing results has not been fully explored. Endothelial dysfunction is accepted as one of the basic pathologic changes in gestational hypertension and preeclampsia/eclampsia. However, it has been argued that these two conditions may differ in etiology and pathology [7]. Preeclampsia is more likely to be involved in severe oxidative DNA damage and placental hypoxic changes than gestational hypertension, while maternal organic vascular disorder is more severe in patients with gestational hypertension [7]. Even among mothers with preeclampsia/eclampsia, the pathological changes in vascular disorders may also vary between early- and late-onset preeclampsia [8]. However, few studies reported the differences between mild and severe gestational hypertension and their relationship to children’s outcomes.

We hypothesized that the severity of hypertension may be a modifier for the effect of hypertensive disorders on birthweight. That is, if hypertension is stratified by severity in further exploratory analysis, it may help to disclose the genesis underlying the opposite findings on birthweight drawn in previous research. In this study, we committed to exploring these associations in two different datasets: the China Labor and Delivery Survey (CLADS) and the US Collaborative Perinatal Project (CPP). Moreover, since the placenta is one of the most important organs in mechanism research for gestational conditions, we further examined the placental pathological changes based on the severity of gestational hypertension in the CPP. We also provided insight into the effect of hypertension severity on children’s blood pressure (BP) and body mass index (BMI) at 7 years of age. Although the two datasets were from different generations and countries, both have comparable information on maternal demographics, concomitants and complications of pregnancy, and the outcomes of children’s birthweight. In addition, even though the prevalence of macrosomia increased in recent decades, its prevalence in contemporary China is not much higher than that in the United States during the 1960s (5.8% vs. 5.5%), making it possible to complete the correlation analysis in these two comparable datasets.

## 2. Materials and Methods

### 2.1. Study Design and Population

**Figure 1:**
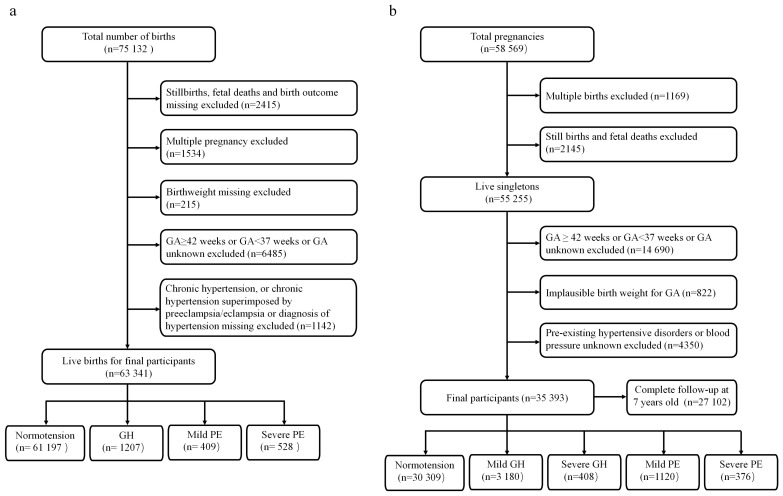
Study flowchart. (**a**) The China Labor and Delivery Survey. (**b**) The United States Collaborative Perinatal Project.

The present study included two cohorts: the CLADS and the CPP. The CLADS, a multicenter cross-sectional study, was conducted from March 2015 to December 2016. Detailed descriptions and protocols about the CLADS were published elsewhere [9]. Finally, 63,341 live-term births were included in the analysis (Figure 1a). The present study was approved by the Ethics Review Board of the Xinhua Hospital Affiliated to the Shanghai Jiao Tong University School of Medicine (XHEC–C–2015–006), the World Health Organization (WHO) Research Ethics Review Committee (HRP Study A65899), and participating hospitals. All methods in the present study were performed according to the Declaration of Helsinki. The present study was an observational, cross-sectional study, and only anonymous clinical information was collected; therefore, consent to participate was deemed unnecessary in accordance with the Ethics Review Board of the Xinhua Hospital Affiliated to the Shanghai Jiao Tong University School of Medicine.

The CPP was a prospective study of the 12 US academic medical centers from 1959 to 1976. About 75% of offspring were followed from birth to 7 years of age. Height/length and weight were measured and recorded at birth, 4 months, and 1, 4, and 7 years of age. Following delivery, placental gross morphology was examined, and samples were collected for histological microscopic examination in accordance with a standard protocol. The CPP data can be found publicly through the US National Archives (https://www.archives.gov/research/electronic-records/nih.html (accessed on 1 September 2021)), and details about the study were published elsewhere [10]. The use of publicly available de-identified data is exempt from review by our Institutional Review Board. Totally, we included 35,393 live births for the final analysis (Figure 1b).

### 2.2. Hypertensive Disorders in Pregnancy

Women in the CLADS were diagnosed with four mutually exclusive categories on the basis of the definitions published in 2013 by the American College of Obstetricians and Gynecologists [11]: normotension, gestational hypertension, mild preeclampsia, or severe preeclampsia/HELLP syndrome (hemolysis, elevated liver enzymes, and low platelets syndrome)/eclampsia. Gestational hypertension was defined as a diastolic blood pressure (DBP) ≥ 90 mmHg or a systolic blood pressure (SBP) ≥ 140 mmHg, or both, on two occasions at least 4 h apart after 20 gestational weeks, but without proteinuria, in a woman with a previously normal BP. Preeclampsia was classified as mild or severe. Mild preeclampsia was defined as DBP ≥ 90 mmHg or SBP ≥ 140 mmHg, measured on at least two occasions after 20 weeks with proteinuria. Severe preeclampsia was defined as DBP ≥ 110 mmHg and/or SBP ≥ 160 mmHg on two occasions at least 4 h apart (unless antihypertensive therapy was initiated before this time), and they developed any of the severe features, including pulmonary edema, renal insufficiency, proteinuria, thrombocytopenia, impaired liver enzyme, visual disturbances, or new-onset headache. Eclampsia is diagnosed by new-onset tonic-clonic, multifocal, or focal seizures in a patient with preeclampsia without other causative conditions, including drug use, infarction, cerebral arterial ischemia, intracranial hemorrhage, or epilepsy. Patients with eclampsia were assigned to the severe preeclampsia group for analysis.

In the CPP, except for women with gestational hypertension, they were further divided into mild and severe depending on the cut-off DBP (110 mmHg). Others were similarly grouped into normotension, mild preeclampsia, and severe preeclampsia/eclampsia (Supplemental Methods S1). Detailed protocols for collecting blood pressure information and classifying hypertensive disorders have been published elsewhere [12].

### 2.3. Outcomes

Macrosomia was determined as a birthweight of >4000 g, and low birthweight (LBW) was <2500 g [13,14]. Large for gestational age (LGA) was determined as birthweight above the 90th percentile for a given gestational age and sex, and small for gestational age (SGA) was below the 10th percentile, according to a fetal-weight reference developed by Hadlock and Gardosi’s notion of proportionality. The generic reference can be easily applied to various populations, provided that the mean birthweight at 40 weeks of gestation in these populations can be captured [15]. In the CPP, long-term outcomes at 7 years of age, including SBP, DBP, and BMI, were treated as continuous and dichotomous variables in the analysis. Height and weight were measured strictly by staff following the standardized CPP procedures [10,16]. Overweight/obesity was defined as a sex- and race-specific BMI above the 85th percentile in the CPP population. BP at 7 years of age was measured using a manual sphygmomanometer on the right arm in a sitting position. SBP and DBP higher than the 90th percentile (internal standard) were considered elevated SBP (>125 mmHg) and elevated DBP (>70 mmHg), respectively [17].

### 2.4. Placental Pathology

In addition, as the CPP was one of the most comprehensive sources of detailed placental pathology, we further examined the differences in placental pathology among various severities of hypertensive disorders, which were presumably involved in the high prevalence of macrosomia in mild gestational hypertension. A detailed method of placental sample collection is described in Supplemental Methods S2, and definitions of placental pathological features are listed in Table S1.

### 2.5. Covariates

Covariates that may potentially affect the exposures and outcomes were considered confounders, if applicable, including maternal age, pre-pregnancy BMI, race, maternal education, socioeconomic status (SES), smoking during pregnancy, marital status, gestational weight gain, gestational age, sex, parity, maternal diseases (preexisting/gestational diabetes, thyroid disease, etc.), and feeding methods. A detailed description is provided in Supplemental Methods S3.

### 2.6. Statistical Analysis

The number of deliveries in every province was provided by the 2016 China Statistical Yearbook [18]. The annual number of births in every province was stratified by hospital level. In the CLADS, every birth was assigned a weight on the basis of inverse probability weighting, considering the number of records reviewed in the hospital at the same hospital level and the number of births in the province at the same hospital level [9]. Univariate and multivariable logistic regression analyses were used to investigate the associations between hypertensive disorders in pregnancy and birth size, controlling for maternal education, race, age, sex, pre-pregnancy BMI, parity, gestational age, and maternal diseases. A sensitivity analysis restricted to mothers without any pre-existing or gestational diabetes, heart, renal, or thyroid diseases was also performed.

In the CPP, the impact of hypertensive disorders in pregnancy on birthweight and long-term outcomes (BMI and BP) at 7 years of age was assessed using multivariable logistic regression and general linear regression models, in which the degree of gestational hypertension was further divided into mild and severe. Moreover, differences in placental pathological measurements among mothers with normotension, mild gestational hypertension, or other severe hypertensive disorders (severe gestational hypertension, mild and severe preeclampsia/eclampsia) were also examined. SAS version 9.4 (SAS Institute Inc., Cary, NC, USA) was used for all analyses.

## 3. Results

### 3.1. Association between Hypertensive Disorders in Pregnancy and Birthweight in the CLADS

**Figure 2:**
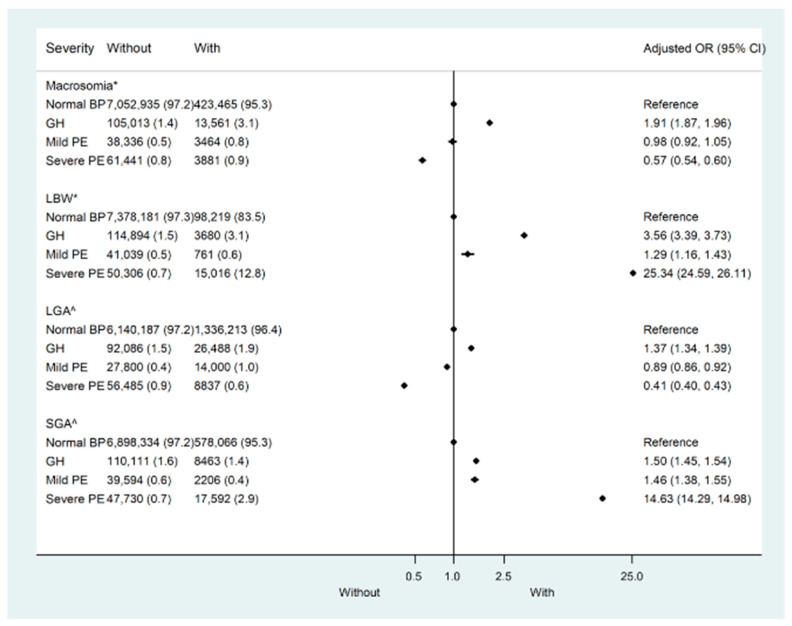
Risk of adverse outcomes by the severity of pregnant hypertensive disorders in the China Labor and Delivery Survey (CLAD). LBW—Low birth weight; LGA—Large for gestational age; SGA—Small for gestational age. * Adjusted for maternal age, race, sex, education, parity, pre-pregnancy body mass index (BMI), heart disease, renal disease, thyroid disease, diabetes mellitus, gestational diabetes, and gestational age. ^^^ Adjusted for maternal age, race, education, parity, pre-pregnancy body mass index (BMI), heart disease, renal disease, thyroid disease, diabetes mellitus, and gestational diabetes.

In the CLADS, the weighted prevalence of macrosomia was 5.8% in total and 5.7%, 11.4%, 8.3%, and 5.9% in mothers with normotension, gestational hypertension, mild preeclampsia, and severe preeclampsia/HELLP syndrome/eclampsia, respectively (Table S2). Compared with mothers with normotension, only those with gestational hypertension were more likely to deliver macrosomia (adjusted OR (aOR): 1.91, 95% CI:1.87–1.96), while a high risk of LBW was found in all hypertensive groups (aOR: 3.56, 95%CI: 3.39–3.73 for gestational hypertension; aOR: 1.29, 95%CI: 1.16–1.43 for mild preeclampsia; and aOR: 25.34, 95%CI: 24.59–26.11 for severe preeclampsia/HELLP syndrome/eclampsia) after adjustment for confounders (Figure 2, Table S3). Similarly, the aOR for LGA was 1.37(95%CI:1.34–1.39) in mothers with gestational hypertension. While aORs for SGA were 1.50(95%CI: 1.45–1.54) in mothers with gestational hypertension, 1.46(95%CI: 1.38–1.55) in mild preeclampsia, and 14.63(95%CI: 14.29–14.98) in severe preeclampsia/HELLP syndrome/eclampsia (Figure 2, Table S3). In the sensitivity analysis, the results remained similar when participants were restricted to mothers without any pre-existing or gestational diabetes or heart, renal, or thyroid diseases (Table S4).

### 3.2. Association between Hypertensive Disorders in Pregnancy and Birthweight in the CPP

**Figure 3:**
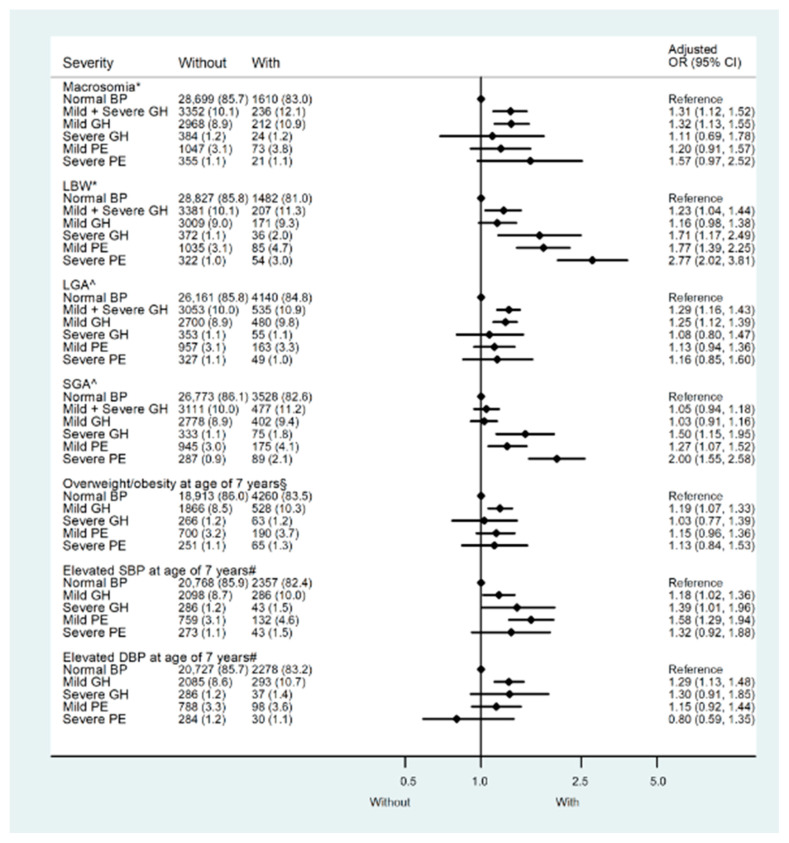
Risk of adverse outcomes by the severity of pregnant hypertensive disorders in the Collaborative Perinatal Project (CPP). LBW: Low birth weight; LGA: Large for gestational age; SGA: Small for gestational age; Elevated SBP: Systolic blood pressure at 7 years of age higher than 125 mmHg, the 90th percentile within the CPP population. Elevated DBP: Diastolic blood pressure at 7 years of age higher than 70 mmHg, the 90th percentile within the CPP population. Overweight/obesity: Race- and sex-specific body mass index at 7 years of age higher than the 85th percentile within the CPP population. GH—Gestational hypertension. Mild PE—mild preeclampsia. Severe PE—Severe preeclampsia/eclampsia. Model *: Adjusted for maternal age, parity, race, sex, maternal education level, marital status, smoking status during pregnancy, socioeconomic status, diabetes, maternal pre-pregnancy body mass index (BMI), gestational weight gain, and gestational age. Model ^: maternal age, parity, race, maternal education level, marital status, smoking status during pregnancy, socioeconomic status, diabetes, maternal pre-pregnancy body mass index (BMI), and gestational weight gain. Model §: Model * + birth weight. Model #: Model * + birth weight and body mass index at 7 years of age.

We conducted similar analyses in the CPP to validate the relationship between hypertensive disorders in pregnancy and birthweight. The baseline characteristics are listed in Tables S5 and S6. The prevalence of macrosomia was 5.5% (1940 of 35,393). As with the results from the CLADS, mothers with gestational hypertension (mild and severe) tended to be associated with both large and small birth sizes. In the further stratified analysis, only those with mild gestational hypertension were more likely to deliver macrosomia (aOR: 1.32; 95% CI:1.13–1.55) and LGA (aOR: 1.25; 95% CI:1.12–1.39), while in mothers with severe hypertensive disorders (severe gestational hypertension, mild and severe preeclampsia/eclampsia), the risks of delivering macrosomia and LGA did not increase. In contrast, except for mothers with mild gestational hypertension, the risk of delivering LBW and SGA babies increased in those with severe hypertensive disorders (Figure 3, Table S7).

When birthweight was treated as a continuous variable, a multivariate general linear regression model confirmed that birthweight was 45.8 g (95%CI: 29.4–62.2) higher in neonates born to mothers with mild gestational hypertension and 89.2 g(95%CI: −135.1–−43.3) lower in neonates born to mothers with severe preeclampsia/eclampsia than those to mothers with normotension, after adjustment for confounders (Table 1). 

### 3.3. Associations between Maternal Hypertensive Disorders in Pregnancy and Childhood Outcomes at 7 Years of Age in the CPP

Compared with children of normotensive mothers, only those whose mothers had mild gestational hypertension had a 1.23-fold (95%CI: 1.11–1.37) increased risk of overweight/obesity at 7 years of age, while children of mothers with severe hypertensive disorders were not at high risk of overweight/obesity (Table S7). Even after the additional adjustment for birthweight, the effect persisted. When BMI was treated as a continuous variable, multivariate general linear regression models also substantiated its relationship with maternal hypertension, and only children of mothers with mild gestational hypertension had significantly higher BMI (adjusted β: 0.03; 95%CI: 0.01–0.05) than those of mothers with normotension (Table 1).

As for childhood BP at 7 years of age, all children from the maternal hypertension groups had higher SBP than those from the normotensive group, ranging from 1.02–1.41 mmHg (Table 1). In the multivariable logistic regression models, high risks of elevated SBP (>125 mmHg) were found in the groups with mild gestational hypertension (aOR: 1.18, 95%CI: 1.02–1.36), severe gestational hypertension (aOR: 1.39, 95%CI: 1.01–1.96), and mild preeclampsia (aOR: 1.58, 95%CI: 1.29–1.94). An increased risk of elevated DBP (>70 mmHg) only existed in the mild gestational hypertension group (aOR: 1.29, 95%CI: 1.13–1.48) after adjustment of confounders (Figure 3, Table S7).

### 3.4. Placental Pathological Features among Mothers with Normotension, Mild Gestational Hypertension, and Other Hypertensive Disorders in the CPP

Since the placenta of hypertensive disorders in pregnancy is always associated with pathological damage and uteroplacental malperfusion, we studied the differences in placental pathology features between mothers with mild (mild gestational hypertension) and severe hypertensive disorders (severe gestational hypertension, mild and severe preeclampsia/eclampsia). Compared with the placenta from normotensive mothers, all those mothers with mild or severe hypertensive disorders tended to have an increased risk of infarct, including old vascular infarct (aOR: 1.24, 95%CI: 1.09–1.41; aOR: 1.81, 95%CI: 1.55–2.11), large infarct size (>3 cm) (aOR: 1.39, 95%CI: 1.13–1.70; aOR: 1.66, 95%CI: 1.28–2.17), and more number of infarcts (aOR: 1.57, 95%CI: 1.38–1.77; aOR: 1.98, 95%CI: 1.69–2.32) (Table 2). The more severe the hypertensive disorder, the higher the risk of vascular lesions. Moreover, in contrast to placenta from mothers with normotension or severe hypertension, those from mothers with mild hypertension were more likely to be mature (≥37 weeks)(aOR: 1.33, 95%CI: 1.11–1.60; aOR: 1.36, 95%CI: 1.04–1.79), at low risk of high placenta-to-birthweight ratio (PBW ratio) (≥90th percentile)(aOR: 0.90, 95%CI: 0.78–1.00; aOR: 0.70, 95%CI: 0.56–0.87), and their chorion villi were more likely to have normal amounts of syncytium-nuclear clumping for term placenta (aOR: 0.73, 95%CI: 0.61–0.86; aOR: 0.63, 95%CI: 0.49–0.80) (Table 2). 

## 4. Discussion

According to the two datasets, mothers with gestational hypertension were more likely to deliver both large and small-birthweight babies. In the CPP, further stratified analysis indicated that only mothers with mild gestational hypertension had an increased risk of macrosomia and LGA, whereas those with severe hypertensive disorders (severe gestational hypertension, mild preeclampsia, and severe preeclampsia/eclampsia) all tended to have LBW and SGA babies. The severity of hypertensive disorders in pregnancy was related to discordant outcomes for the offspring.

This discrepancy in neonatal size may be attributed to the severity of hypertensive disorders. The proportion of different severities of gestational hypertension among previous studies presumably led to opposite results and may account for the aforementioned opposing arguments in the Introduction section [5,6]. Preeclampsia is currently classified as early-onset, linked to poor placentation and fetal growth restriction, whereas late-onset is suggested to result from maternal factors, and both may be associated with placental syncytiotrophoblast stress. Late-onset preeclampsia is proposed as a predisposing inflammatory status that may result in an abnormal response of maternal vessels to the “stress of pregnancy” [19]. This should be considered if there is no poor spiral artery remodeling. Since all participants in the present study were term deliveries, late-onset pathogenesis probably played a major role. Owaki et al. confirmed that the placenta of mothers with preeclampsia/eclampsia presented apparent hypoxic changes [7], including upregulation of an antiangiogenic factor (sFlt-1), oxidative deoxyribonucleic acid (DNA) damage, and downregulation of an angiogenic factor (PlGF). They also reported that these changes are less significant in mothers with gestational hypertension than in mothers with normotension [7]. These findings may be associated with LBW and SGA babies in mothers with severe gestational hypertension and preeclampsia/eclampsia; however, they may not explain macrosomia and LGA in mothers with mild gestational hypertension. There must be other pathogens as well.

To investigate the potential pathological mechanisms related to macrosomia and LGA, we provided insight into the pathological features of the placenta. Given that mothers with mild gestational hypertension had a higher risk of giving birth to macrosomia and LGA than mothers with normotension and severe hypertensive disorders, only placenta features that were significantly in discord with the other two groups in the same direction could be considered as potential pathological pathogenesis related to macrosomia and LGA. Therefore, three placental features, the placenta-to-birthweight ratio (PBWratio), placental maturity, and syncytium-nuclear clumping, were identified. The PBW ratio is a commonly used indicator of the ability of the placenta to maintain adequate nutrient supply to the fetus, and a high ratio is often described as “placental dysfunction” [20]. Gestational hypertensive disorders have significant effects on the growth of both placentas and fetuses. In a Brazilian birth cohort, researchers reported a higher PBW ratio in mothers with preeclampsia compared with normotensive mothers but no difference from those with gestational hypertension. They, however, did not stratify the severity of gestational hypertension into mild and severe groups, which may cover up their different impacts on placental pathology [21]. Syncytium-nuclear clumping, i.e., syncytial knotting, increases with increasing gestational age and is a typical feature of terminal villi in the placenta [22]. Much syncytium-nuclear clumping in term placental syncytial knots is associated with conditions of uteroplacental malperfusion, while less may imply a relatively well-developed placenta. Placental maturity above 37 weeks is a subjective indicator that comes with the CPP database and was defined by the pathologists in that era based on comprehensive considerations of several relevant pathological measurements. All three features indicated a relatively mature placenta in the mild gestational hypertension group, even more than in normotensive mothers, suggesting pathological mechanisms related to macrosomia and LGA in mild gestational hypertension, or at least of a relational nature. Further exploration of the potential pathogenesis of these pathological changes is required.

Regarding childhood BP at the age of 7 years, all children, except those from the severe preeclampsia/eclampsia group, had an increased risk of elevated SBP (>125 mmHg), and the more severe the gestational hypertensive disorder, the higher the risk of elevated SBP in childhood. In contrast, the high risk of elevated DBP (>70 mmHg) was found only in mild gestational hypertension. The pathogenesis of high BP in mild gestational hypertension may not be the same as that in the severe hypertensive groups. Systolic hypertension stems from reduced distensibility of major arteries and is often diagnosed in elderly individuals, while diastolic hypertension is more likely to be identified in the middle-aged population with various etiologies. During the last three decades, accumulating epidemiological studies have emphasized systolic, not diastolic, pressure, which is the most important indicator of cardiovascular risk and mortality [23]. Hozawa et al. confirmed that the cardiovascular event rate of isolated systolic hypertension is almost 8-fold higher than that of isolated diastolic hypertension (2.04 vs. 0.26 deaths per 100 person-years) [24]. Although these conclusions were derived from adult cohorts, they, in a sense, may suggest that children from the mild gestational hypertension group had an increased risk of overweight/obesity but relatively milder cardiovascular lesions than those from the severe groups.

In an earlier study, authors reported that offspring exposed to preeclampsia could be causally associated with later hypertension, which was no longer significant after controlling for offspring BMI [25]. They argued that exposure to preeclampsia could be causally associated with later hypertension, which may be confounded by the offspring’s higher BMI. Our study indicated that the relationship between hypertensive disorders and childhood outcomes was independent of overweight/obesity, as it persisted after controlling for confounders including birth weight and overweight/obesity at 7 years old. Intrauterine exposure to hypertensive disorders was possibly a risk factor for elevated blood pressure in childhood. A few studies on childhood hypertension, especially on the difference between systolic and diastolic hypertension in children, have been reported [26].

Our study has some limitations. First, gestational hypertension was not divided into mild and severe in the CLADS, hampering the exploration of the association between mild gestational hypertension and birthweight. However, the use of the data from the CPP compensated for the deficiency. Second, although the CPP was done several decades ago and had its weaknesses, it is still one of the largest maternal-birth datasets, including maternal characteristics and a 7-year follow-up of child growth. It also provided the most comprehensive source for a detailed prospective placental database. Well-trained pathologists examined placental pathology rigorously according to the protocol in a way that was blinded to the clinical course, making the data more reliable. Third, an inherent problem in studying the placenta is that placental examination is cross-sectional, but pregnancy is longitudinal; thus, we cannot know when these lesions have formed. Fourth, we adjusted for gestational weight gain in the CPP models; however, it was not available in the CLADS. Also, we did not have information on type of treatment or adherence to treatment in the CLADS; thus, their confounding and independent effects are unclear. Lastly, two cohorts were used in the study, one for the main results and the other for replication. This can reduce the false-positive estimates and enhance the analysis power, even though generational differences could be a concern, e.g., the change in definition of hypertension disorders and medical protocol standards. The ACOG guideline released in 2013 chose to continue using the classification schema first introduced in 1972, although there were limited modifications [27]. In the absence of proteinuria, preeclampsia can currently be diagnosed if hypertension is complicated by any of the severe features mentioned in the Methods section. Two, the diagnostic time moved forward to 20 gestational weeks. Nevertheless, neither may have obvious effects on the definition of mild gestational hypertension, as the latter theoretically occurs during late pregnancy [19].

## 5. Conclusions

We confirmed that the severity of hypertensive disorders in pregnancy was related to discordant children’s outcomes. Moreover, several placental pathological features identified in this study may partially account for these discordant outcomes and may be helpful for further research on these findings.

## Figures and Tables

**Table 1:** Polynomial comparison between adverse outcomes at 7 years of age and pregnant hypertensive disorders, the Collaborative Perinatal Project.

			Crude β	P	Adjusted β ^a^	P	Adjusted β ^b^	P
		Mean (SD)	(95% CI)		(95% CI)		(95% CI)	
Birth weight (g)	Normal BP	3243 (464)	Ref	Ref	Ref	Ref		
	Mild GH	3287 (487)	43.9 (26.7, 61.0)	<0.0001	45.8 (29.4, 62.2)	<0.0001		
	Severe GH	3195 (534)	−48.1 (−94.0, −2.1)	0.040	−16.8 (−60.8, 27.3)	0.456	/	/
	Mild PE	3243 (512)	−0.40 (−28.4, 27.7)	0.980	−5.2 (−32.0, 21.6)	0.703		
	Severe PE	3084 (592)	−159.1 (−206.9, −111.2)	<0.0001	−89.2 (−135.1, −43.3)	<0.0001		
BMI (kg/m^2^)	Normal BP	16.0 (1.9)	Ref	Ref	Ref	Ref	Ref	Ref
	Mild GH	16.2 (2.1)	0.26 (0.17, 0.34)	<.0001	0.22 (0.13, 0.30)	<0.0001	0.03 (0.01, 0.05)	0.001 *
	Severe GH	15.9 (2.0)	−0.07 (−0.29, 0.14)	0.498	−0.07 (−0.29, 0.15)	0.550	0.01 (−0.04, 0.05)	0.786 *
	Mild PE	16.1 (2.1)	0.15 (0.02, 0.28)	0.029	0.11 (−0.02, 0.25)	0.097	0.02 (−0.01, 0.05)	0.108 *
	Severe PE	16.1 (2.1)	0.10 (−0.12, 0.32)	0.359	0.10 (−0.13, 0.32)	0.393	0.02 (−0.02, 0.07)	0.343 *
SBP (mmHg)	Normal BP	101.8 (10.1)	Ref	Ref	Ref	Ref	Ref	Ref
	Mild GH	102.9 (10.3)	1.16 (0.74, 1.59)	<0.0001	1.22 (0.78, 1.66)	<0.0001	1.02 (0.59, 1.45)	<0.0001
	Severe GH	102.9 (10.5)	1.11 (0.01, 2.22)	0.049	1.25 (0.11, 2.40)	0.032	1.26 (0.15, 2.38)	0.026
	Mild PE	103.2 (10.6)	1.42 (0.74, 2.11)	<0.0001	1.49 (0.79, 2.19)	<0.0001	1.41 (0.72, 2.09)	<0.0001
	Severe PE	103.3 (10.9)	1.53 (0.40, 2.66)	0.008	1.32 (0.15, 2.49)	0.027	1.33 (0.19, 2.47)	0.022
DBP (mmHg)	Normal BP	61.4 (9.6)	Ref	Ref	Ref	Ref	Ref	Ref
	Mild GH	62.2 (9.7)	0.86 (0.45, 1.26)	<0.0001	0.85 (0.43, 1.27)	<0.0001	0.70 (0.29, 1.12)	0.001
	Severe GH	61.3 (10.4)	−0.09 (−1.14, 0.97)	0.873	−0.02 (−1.12, 1.07)	0.959	−0.06 (−1.15, 1.02)	0.901
	Mild PE	61.2 (10.2)	−0.10 (−0.75, 0.54)	0.751	−0.20 (−0.87, 0.47)	0.531	−0.20 (−0.87, 0.46)	0.526
	Severe PE	61.3 (10.0)	−0.08 (−1.15, 1.00)	0.891	−0.12 (−1.23, 0.99)	0.820	−0.15 (−1.26, 0.95)	0.769

BP—Blood pressure.GH—Gestational hypertension. Mild PE—mild preeclampsia. Severe PE—Severe preeclampsia/eclampsia. BMI—Body mass index measured at 7 years of age. SBP—Systolic blood pressure measured at 7 years of age. DBP—Diastolic blood pressure measured at 7 years of age. Model ^a^: Adjusted for parity, diabetes, maternal education level, race, sex, marital status, smoking status during pregnancy, socioeconomic status, maternal pre-pregnancy body mass index (BMI), gestational weight gain, and gestational age. Model ^b^: Model a + birth weight and BMI at 7 years of age. * Model ^a^ + birth weight.

**Table 2:** Association between placental pathology lesions and pregnant hypertensive disorders, the Collaborative Perinatal Project.

	Control Group	Mild Group	Severe Group	Mild Group vs. Control Group	Mild Group vs. Control Group	Severe Group vs. Control Group	Severe Group vs. Control Group	Mild Group vs. Severe Group	Mild Group vs. Severe Group
	*N* (%)	*N* (%)	*N* (%)	Crude OR	Adjusted OR ^a^	Crude OR	Adjusted OR ^a^	Crude OR	Adjusted OR ^a^
*N*(%)	30286 (85.6)	3177 (9.0)	1904 (5.4)	/	/	/	/	/	/
PBW ratio ≥90th percentile *	2486 (9.7)	238 (8.5)	185 (11.7)	0.86 (0.75, 0.98)	0.90 (0.78, 1.00)	1.23 (1.05, 1.45)	1.28 (1.08, 1.51)	0.70 (0.57, 0.85)	0.70 (0.56, 0.87)
Placental maturity ≥37 weeks	23793 (92.8)	2668 (94.6)	1479 (93.3)	1.38 (1.16, 1.63)	1.33 (1.11, 1.60)	1.08 (0.88, 1.32)	1.01 (0.82, 1.25)	1.28 (1.00, 1.65)	1.36 (1.04, 1.79)
Abruptio placenta	369 (1.2)	40 (1.3)	24 (1.3)	1.02 (0.74, 1.42)	1.10 (0.78, 1.55)	1.02 (0.68, 1.55)	1.27 (0.81, 1.97)	1.00 (0.60, 1.66)	0.92 (0.53, 1.58)
Calcification	3207 (12.5)	348 (12.4)	227 (14.3)	0.99 (0.88, 1.11)	0.91 (0.80, 1.04)	1.17 (1.01, 1.35)	0.99 (0.85, 1.17)	0.84 (0.70, 1.01)	0.91 (0.74, 1.10)
Vascular lesions									
Old vascular infarct	2202 (8.6)	317 (11.3)	234 (14.7)	1.35 (1.19, 1.53)	1.24 (1.09, 1.41)	1.84 (1.59, 2.13)	1.81 (1.55, 2.11)	0.73 (0.61, 0.88)	0.68 (0.56, 0.82)
Large size of infarct	798 (3.1)	119 (4.2)	72 (4.6)	1.37 (1.13, 1.67)	1.39 (1.13, 1.70)	1.48 (1.16, 1.90)	1.66 (1.28, 2.17)	0.93 (0.69, 1.25)	0.82 (0.60, 1.13)
Number of infarcts	2154 (8.4)	382 (13.6)	236 (14.9)	1.71 (1.52, 1.92)	1.57 (1.38, 1.77)	1.91 (1.66, 2.21)	1.98 (1.69, 2.32)	0.89 (0.75, 1.07)	0.78 (0.65, 0.94)
Vessel thrombosis	181 (0.7)	27 (1.0)	8 (0.5)	1.36 (0.91, 2.04)	1.33 (1.14, 1.54)	0.71 (0.35, 1.45)	1.43 (1.17, 1.74)	1.91 (0.86, 4.21)	0.90 (0.71, 1.15)
Vessel atheroma	101 (0.4)	9 (0.3)	4 (0.3)	0.81 (0.41, 1.60)	0.73 (0.36, 1.45)	0.64 (0.24, 1.75)	0.53 (0.19, 1.46)	1.26 (0.39, 4.08)	1.34 (0.40, 4.53)
Villous lesions									
Less syncytium-nuclear clumping for term placenta	2196 (8.7)	170 (6.2)	150 (9.7)	0.69 (0.59, 0.81)	0.73 (0.61, 0.86)	1.12 (0.94, 1.34)	1.15 (0.96, 1.39)	0.61 (0.49, 0.77)	0.63 (0.49, 0.80)
Much syncytium-nuclear clumping for term placenta	465 (2.0)	67 (2.5)	41 (2.9)	1.28 (0.99, 1.66)	1.28 (0.98, 1.67)	1.45 (1.05, 2.01)	1.65 (1.18, 2.30)	0.89 (0.60, 1.31)	0.82 (0.55, 1.23)
Villous infarction	3528 (13.8)	396 (14.1)	331 (20.9)	1.03 (0.92, 1.15)	1.03 (0.91, 1.15)	1.65 (1.46, 1.88)	1.61 (1.40, 1.84)	0.62 (0.53, 0.73)	0.63 (0.53, 0.74)
Stromal fibrosis	193 (0.8)	31 (1.1)	16 (1.0)	1.47 (1.01, 2.15)	1.51 (1.01, 2.25)	1.34 (0.80, 2.24)	1.47 (0.85, 2.53)	1.09 (0.60, 2.01)	1.04 (0.55, 1.96)
Villous cytotrophoblast layer	120 (0.5)	11 (0.4)	8 (0.5)	0.83 (0.45, 1.55)	0.86 (0.45, 1.66)	1.08 (0.53, 2.21)	1.19 (0.57, 2.48)	0.77 (0.31, 1.93)	0.77 (0.30, 2.00)
Cord membranous insertion	387 (1.5)	38 (1.4)	39 (2.5)	0.89 (0.64, 1.25)	0.95 (0.77, 1.16)	1.64 (1.18, 2.29)	1.13 (0.86, 1.49)	0.54 (0.35, 0.85)	0.88 (0.64, 1.21)
Inflammatory cell infiltration									
Neutrophilic infiltration	2231 (8.7)	278 (9.9)	157 (9.9)	1.15 (1.01, 1.31)	1.11 (0.97, 1.28)	1.15 (0.97, 1.37)	1.04 (0.86, 1.25)	1.00 (0.81, 1.23)	1.13 (0.90, 1.40)
Lymphocytic infiltration	254 (1.0)	32 (1.1)	20 (1.3)	1.15 (0.79, 1.66)	1.21 (0.82, 1.78)	1.28 (0.81, 2.02)	1.28 (0.79, 2.07)	0.90 (0.51, 1.58)	1.03 (0.57, 1.87)

Control group: Mothers without any preexisting or gestational hypertensive disorders. Mild group: Mothers with mild gestational hypertension. Severe group: Mothers with severe gestational hypertension, mild preeclampsia, severe preeclampsia, or eclampsia. * Placenta-to-birth weight ratio higher than the 90th percentile in the CPP. ^a^ Adjusted for maternal age, maternal ethnicity, parity, socioeconomic status, maternal education, marital status, maternal smoking history, pre-pregnancy body mass index (BMI), gestational weight gain, diabetes, gestational age, and sex.

## Data Availability

The CPP data can be found publicly through the US National Archives (https://www.archives.gov/research/electronic-records/nih.html (accessed on 1 September 2021)). The CLADS data will be made available upon reasonable request to the corresponding author (Y.Z.).

## Supplementary Materials

The following supporting information can be downloaded at: https://www.mdpi.com/article/10.3390/nu15143104/s1. Additional information is included in this study [28,29,30,31,32,33]: Supplemental Methods S1: Definition of hypertensive disorders in the CPP; Supplemental Methods S2: Placental sample collection; Supplemental Methods S3: Definitions of covariates; Table S1. Definition of placental pathological lesions, the Collaborative Perinatal Project; Table S2. Distribution of characteristics, the China Labor and Delivery Survey, 2015–2016; Table S3. Associations of hypertensive disorders in pregnancy with birth size: China, 2015–2016; Table S4. Sensitivity analysis restricted to mothers without any pre-existing or gestational diabetes or heart, renal, or thyroid diseases, the China Labor and Delivery Survey, 2015–2016.Table S5. Baseline characteristics by macrosomia, the collaborative perinatal project. Table S6. Baseline characteristics by large gestational age, the collaborative perinatal project. Table S7. Risk ratio between adverse outcomes at 7 years of age and pregnant hypertensive disorders, the Collaborative Perinatal Project.
