# Supplementary material for: Association of the Severity of Hypertensive Disorders in Pregnancy with Birthweight, Childhood Obesity, and Blood Pressure at Age 7
Source: Nutrients. 2023 Jul 11;15(14):3104. doi: 10.3390/nu15143104 (PMC10384928; doi:10.3390/nu15143104)
Supplement: Supplementary file 1 [file nutrients-15-03104-s001.zip › nutrients-2467080-supplementary.pdf]

Supplemental methods

1. *Definition of hypertensive disorders in the CPP*

Mild hypertension: diastolic blood pressure ≥ 90 mmHg but < 110 mmHg;

Severe hypertension: diastolic blood pressure ≥ 110 mmHg;

Mild proteinuria: urinary albumin 1+ or 2+;

Severe proteinuria: urinary albumin >= 3+;

Gestational hypertension: mild hypertension on two occasions or severe hypertension on one occasion from gestational age 24 weeks to 2 weeks postpartum;

Gestational proteinuria: mild proteinuria on two occasions or severe proteinuria on one occasion from gestational age 24 weeks to 2 weeks postpartum;

Mild gestational hypertension: mild hypertension alone;

Severe gestational hypertension: severe hypertension alone;

Mild preeclampsia: mild gestational hypertension + gestational proteinuria;

Severe preeclampsia and eclampsia: severe gestational hypertension plus any of the following symptoms: gestational proteinuria, oliguria, pulmonary edema, or convulsion from 24 weeks of gestation to 2 weeks postpartum;

2. *Placental sample collection*

According to a standardized protocol by Dr Naeye [1], pathologists first conducted a gross examination of the freshly delivered placentas, followed by taking representative placental blocks from the central portion. In addition, one membrane roll sample, one umbilical cord sample, and samples from apparent gross

abnormalities were also obtained for microscopic examination. Pathologists conducting the placental examination were blinded to the clinical course for 98% of the gross and 97% of the microscopic examinations. Pathological lesions of the placenta were defined as dichotomous variables with one or more lesions based on the literature [2,3].

3. Definitions of covariates

In the China Labor and Delivery Survey (CLADS), maternal pre-pregnancy body mass index (BMI) was defined as weight in kilograms divided by the square of height in meters (kg/m^2) and was categorized as follows: underweight (<18.5 kg/m^2), normal (18.5–24.9 kg/m^2), and overweight or obesity (≥25 kg/m^2) according to the WHO BMI classification [4]. Maternal medical conditions included pre-existing/gestational diabetes and heart, renal, and thyroid diseases. The gestational age (GA) was ascertained based on the last menstrual period (LMP) or ultrasound dating in the first trimester when the date of the last menstrual period was uncertain. Maternal education levels were categorized as low (illiterate, primary school, and junior school), middle (high school, technical school, and junior college), and high (college or higher).

In the United States (US) Collaborative Perinatal Project (CPP), as GA was calculated only based on the LMP, pregnancies with implausible combinations of birth weight and GA were excluded [5]. Other covariables consisted of pre-pregnancy BMI [underweight (<18.5 kg/m^2), normal (18.5 – 24.9 kg/m^2), and overweight/obesity (≥25 kg/m^2)], maternal age (<20, 20–24, 25–29, 30–35, and ≥35 years), race (White, Black,

and other), parity (0, 1, and ≥2), socioeconomic status [socioeconomic status (SES), 1-5 grades from the lowest to the highest) [6], maternal education (<10, 10–12, and >12 years), marital status (no or yes), smoking during pregnancy (no or yes), gestational weight gain (<10.0, 10.0–14.9, and ≥15.0kg), and feeding methods (exclusively breast, exclusively bottle, and mixed feed).

Reference

1. Naeye RL. Effects of maternal cigarette smoking on the fetus and placenta. Br J Obstet Gynaecol. 1978;85:732–7.
2. Ghidini A, Salafia CM, Pezzullo JC. Placental vascular lesions and likelihood of diagnosis of preeclampsia. Obstet Gynecol 1997;90(4 Pt 1):542–5.
3. Redline RW, Heller D, Keating S, Kingdom J. Placental diagnostic criteria and clinical correlation−a workshop report. Placenta 2005;26 Suppl A:S114−7.
4. WHO Expert Consultation. Appropriate body-mass indexfor Asian populations and its implicationsfor policy and intervention strategies. Lancet. 2004;363:157–163.
5. Alexander GR, Himes JH, Kaufman RB, Mor J, Kogan M. A United States national reference for fetal growth. Obstet Gynecol 1996; 87: 163-168.
6. Myrianthopoulos NC, French KS. An application of the U.S. Bureau of the Census socioeconomic index to a large, diversified patient population. Soc Sci Med. 1968; 2: 283-299.

Table S1. Definition of placental pathological lesions, the Collaborative Perinatal Project.

Placental lesions*	Definition
**PBW ratio ≥90thpercentile**	Placental weight and birth weight ratio higher than 90thpercentile for a given gestational week
**Placental maturity**	Placental maturity above37 gestational weeks
**Abruptio placenta**	Placenta detach from its decidual site
**Calcification**	Calcification throughout cut surface
**Vascular lesions**	
Old vascular infarct	Yellow infarct in maternal surface, not marginal
Size of infarct	At least one infarct ≥ 3 cm in cut surface
Number of infarcts	Number of infarcts≥ 3
Vessel thrombosis	Vessel thrombosis in cord, membrance, chorionic plate and cutsurface
Vessel atheroma	Vessel atheroma in decidua
**Villous lesions**	
Less syncytium-nuclear clumping	Syncytium-nuclear clumping less than normal for term placenta in decidua
Much syncytium-nuclear clumping	Syncytium-Nuclear clumping much than normal for term placenta in decidua
Villous infarct	Micro infarcts in terminal villi or intervillious thrombi with adjacent villous infarct
Stromal fibrosis	Many stromal fibrosis presented in terminal villi
Villous cytotrophoblast layer	Occurrence of villous cytotrophoblast layer
**Membranous insertion of cord**	Membranous insertion
**Inflammatory cell infiltration**	
Neutrophilic infiltration	Neutrophilic infiltration in decidua, chorion or amnion of membrane roll, chorion of placental surface, umbilical vessels or cord substance
Lymphocytic infiltration	Lymphocytic infiltration in capsularis, basalis or at margin

* Each placental lesion was transformed into a dichotomous variable, coded as either “0” for negative or “1” for positive pathologic findings.

Table S2. Distribution of characteristics, the China Labor and Delivery Survey, 2015-2016

Characteristics	Weighted number of live births (%) n = 7702096	Weighted number of macrosomia (per 100 live births)	Weighted number of LBW (per 100 live births)	Weighted number of LGA (per 100 live births)	Weighted number of SGA (per 100 live births)
**Maternal age**					
≤ 19	176245 (2.3)	2333 (1.3)	9943 (5.6)	9462 (5.4)	25566 (14.5)
20-35	6933895 (90.0)	377546 (5.4)	93734 (1.4)	1212069 (17.5)	537187(7.7)
≥36	591957 (7.7)	64491 (10.9)	13998 (2.4)	164006 (27.7)	43574 (7.4)
**Race**					
Han	7446523 (96.7)	423054 (5.7)	109998 (1.5)	1341165 (18.0)	583413 (7.8)
Other	255573 (3.3)	21316 (8.3)	7678 (3.0)	43372 (17.4)	22914 (9.0)
**Mother's education level** [a]					
Low	2582929 (37.3)	156937 (6.1)	41147 (1.6)	436446 (16.9)	241287 (9.3)
Middle	2724124 (39.3)	164480 (6.0)	46479 (1.7)	518103 (19.0)	196229 (7.2)
High	1626923 (23.5)	85960 (5.3)	12696 (0.8)	297274 (18.3)	106991 (6.6)
**Pre-pregnancy BMI**					
Underweight (<18.5 kg/m^2)	721145 (13.7)	13283 (1.8)	18163 (2.5)	65689 (9.1)	100416 (13.9)
Normal (18.5–24.9 kg/m^2)	3793252 (72.3)	218852 (5.8)	43520 (1.1)	686403 (18.1)	249932 (6.6)
Overweight or obesity (≥25 kg/m^2)	732927 (14.0)	96021 (13.1)	10631 (1.5)	215249 (29.4)	26905 (3.7)
**Parity**					
0	2564093 (33.4)	123518 (4.8)	39176 (1.5)	360831 (14.1)	254403 (9.9)
≥1	5117475 (66.6)	320285 (6.3)	78308 (1.5)	1021935 (20.0)	351381 (6.9)
**Diabetes mellitus**					
No	7578855 (99.2)	428761 (5.7)	114711 (1.5)	1346147 (17.8)	591064 (7.8)
Yes	63114 (0.8)	13607 (21.6)	1491 (2.4)	30407 (48.2)	3785 (6.0)
**Heart disease**					
No	7661497 (99.7)	443281 (5.8)	116868 (1.5)	1376476 (18.0)	602950 (7.9)
Yes	24672 (0.3)	735 (3.0)	502 (2.0)	6509 (26.4)	1702 (6.9)
**Renal disease**					
No	7680641 (99.9)	443114 (5.8)	117202 (1.5)	1378793 (18.0)	604644 (7.9)
Yes	8988 (0.1)	216 (2.4)	191 (2.1)	4759 (52.9)	494 (5.5)
**Thyroid disease**					
No	7289449 (97.0)	419585 (5.8)	113054 (1.6)	1312619 (18.0)	571418 (7.8)

Yes	222479 (3.0)	9058 (4.1)	2775 (1.2)	36231 (16.3)	18787 (8.4)
**Hypertensive disorders in pregnancy**					
No	7476400 (97.1)	423465 (5.7)	98219 (1.3)	1336213 (17.9)	578066 (7.7)
Gestational hypertension	118574 (1.5)	13561 (11.4)	3680 (3.1)	26488 (22.3)	8463 (7.1)
mild preeclampsia	41800 (0.5)	3464 (8.3)	761 (1.8)	14000 (33.5)	2206 (5.3)
Severe preeclampsia/HELLP/ eclampsia	65322 (0.8)	3881 (5.9)	15016 (23.0)	8837 (13.5)	17592 (26.9)
**Gestational diabetes**					
No	6661182 (89.6)	357372 (5.4)	105683 (1.6)	1140825 (17.1)	526617 (7.9)
Yes	773880 (10.4)	81792 (10.6)	4243 (0.5)	222044 (28.7)	41935 (5.4)
**Sex**					
Female	3605022 (46.8)	153456 (4.3)	60227 (1.7)	631383 (17.5)	325395 (9.0)
Male	4093900 (53.2)	290314 (7.1)	57416 (1.4)	754154 (18.4)	280933 (6.9)

LBW: Low birth weight.
LGA: Large for gestational age.
SGA: Small for gestational age.
[a] Maternal education level: low (illiterate, primary school, and junior school), middle (high school, technical school, and junior college), and high (college or higher degree)
BMI, body mass index.

Table S3. Associations of hypertensive disorders in pregnancy with birth size: China, 2015-2016

Hypertensive disorders in pregnancy	Macrosomia (>4000g)		LBW (<2500g)		LGA (>90th percentile)		SGA (<10th percentile)	
	Crude OR (95% CI)	Adjusted OR (95% CI) [a]	Crude OR (95% CI)	Adjusted OR (95% CI)[a]	Crude OR (95% CI)	Adjusted OR (95% CI)[b]	Crude OR (95% CI)	Adjusted OR (95% CI)[b]
No	Ref	Ref	Ref	Ref	Ref	Ref	Ref	Ref
Gestational hypertension	2.15 (2.11, 2.19)	1.91 (1.87, 1.96)	2.41 (2.33, 2.49)	3.56 (3.39, 3.73)	1.32 (1.31, 1.34)	1.37 (1.34, 1.39)	0.92 (0.90, 0.94)	1.50 (1.45, 1.54)
Mild preeclampsia	1.51 (1.45, 1.56)	0.98 (0.92, 1.05)	1.39 (1.30, 1.50)	1.29 (1.16, 1.43)	2.31 (2.27, 2.36)	0.89 (0.86, 0.92)	0.67 (0.64, 0.69)	1.46 (1.38, 1.55)
Severe preeclampsia/HELLP/eclampsia	0.98 (0.95, 1.02)	0.57 (0.54, 0.60)	22.42 (21.99, 22.86)	25.34 (24.59, 26.11)	0.72 (0.71, 0.74)	0.41 (0.40, 0.43)	4.44 (4.36, 4.51)	14.63 (14.29, 14.98)

LBW: Low birth weight.

LGA: Large for gestational age.

SGA: Small for gestational age.

[a]Adjusted for maternal age, race, education, sex, parity, pre-pregnancy body mass index (BMI), heart disease, renal disease, thyroid disease, diabetes mellitus, gestational diabetes, and gestational age.

[b]Adjusted for maternal age, race, education, parity, pre-pregnancy body mass index (BMI), heart disease, renal disease, thyroid disease, diabetes mellitus, and gestational diabetes.

Table S4. Sensitivity analysis restricted to mothers without any pre-existing or gestational diabetes or heart, renal, or thyroid diseases, the China Labor and Delivery Survey, 2015-2016.

Hypertensive disorders in pregnancy	Macrosomia		LGA		LBW		SGA	
	Crude OR (95% CI)	Adjusted OR (95%CI)[a]	Crude OR (95% CI)	Adjusted OR (95%CI) [b]	Crude OR (95% CI)	Adjusted OR (95%CI)[a]	Crude OR (95% CI)	Adjusted OR (95%CI)[b]
No	Ref	Ref	Ref	Ref	Ref	Ref	Ref	Ref
Gestational hypertension	1.85 (1.81, 1.89)	1.64 (1.60, 1.69)	1.11 (1.09, 1.13)	1.26 (1.23, 1.29)	1.77 (1.69, 1.84)	1.80 (1.67, 1.93)	0.84 (0.82, 0.86)	1.03 (1.01, 1.05)
Mild preeclampsia	1.56 (1.50, 1.62)	1.02 (0.97, 1.08)	2.47 (2.42, 2.53)	0.92 (0.88, 0.95)	1.40 (1.30, 1.52)	1.07 (1.01, 1.15)	0.76 (0.73, 0.80)	1.56 (1.47, 1.65)
Severe preeclampsia/HELLP/ eclampsia	0.88 (0.84, 0.91)	0.93 (0.90, 0.97)	0.71 (0.69, 0.72)	0.71 (0.69, 0.74)	26.20 (25.67, 26.74)	27.35 (26.50, 28.23)	5.09 (4.99, 5.19)	15.80 (15.41, 16.19)

LBW: Low birth weight.
LGA: Large for gestational age.
SGA: Small for gestational age.
[a]Adjusted for maternal age, race, education, parity, pre-pregnancy BMI, sex, and gestational age
[b]Adjusted for maternal age, race, education, parity, and pre-pregnancy BMI.

Table S5. Baseline characteristics by macrosomia, the collaborative perinatal project.

	Without macrosomia N (%)	With macrosomia N (%)	χ^2	p
N (%)	33453 (94.5)	1940 (5.5)		
**Maternal age (years)**			230.3	***<.0001***
< 20	7924 (23.7)	257 (13.3)		
20–24	12719 (38.0)	671 (34.5)		
25–29	7246 (21.7)	476 (24.6)		
30–34	3981 (11.9)	366 (18.9)		
≥ 35	1583 (4.7)	169 (8.7)		
Mean (SD)	23.9 (5.7)	26.0 (6.1)	-16.0	***<.0001***
**Race**			315.6	***<.0001***
White	16939 (50.6)	1383 (71.3)		
Black	13970 (41.8)	454 (23.4)		
other	2544 (7.6)	103 (5.3)		
**Parity**			226.8	***<.0001***
0	10797 (32.3)	371 (19.1)		
1	8011 (24.0)	394 (20.3)		
≥ 2	14621 (43.7)	1174 (60.6)		
**SES**			58.8	***<.0001***
1 (Lowest)	2283 (7.0)	90 (4.8)		
2	9611 (29.6)	472 (25.1)		
3	9992 (30.8)	564 (30.0)		
4	6837 (21.1)	471 (25.0)		
5 (Highest)	3729 (11.5)	284 (15.1)		
**Maternal education levels (year)**			14.0	***<.0001***
Less than high school (≤9)	9508 (28.5)	502 (26.0)		
High school (10–12)	19389 (58.1)	1120 (57.9)		
College and above (>12)	4464 (13.4)	311 (16.1)		
**Married**	25788 (77.1)	1639 (84.5)	57.5	***<.0001***
**Smoking during pregnancy**	15708 (47.3)	654 (34.1)	127.4	***<.0001***
**Pre-pregnant BMI (kg/m2)**			247.7	***<.0001***
<18·5	5015 (15.3)	189 (9.9)		
18·5–24.9	21848 (66.4)	1113 (57.9)		
>25.0	6023 (18.3)	617 (32.2)		
Mean (SD)	22.1 (6.9)	23.9 (7.9)	-11.3	***<.0001***
**Gestational weight gain (kg)**			93.7	***<.0001***
< 10.0	1799 (5.4)	57 (3.0)		
10.0 – 14.9	4467 (13.4)	138 (7.1)		
≥ 15.0	26977 (81.2)	1739 (89.9)		
Mean (SD)	11.9 (8.0)	13.9 (7.7)	10.8	***<.0001***
**Pre-existed or gestational diabetes**	518 (1.6)	86 (4.5)	91.0	***<.0001***
**Sex**			145.4	***<.0001***
Male	16651 (49.8)	1239 (63.9)		
Female	16794 (50.2)	701 (36.1)		

BMI: body mass index
SES: socioeconomic status

Table S6. Baseline characteristics by large for gestational age, the collaborative perinatal project.

	Without LGA N (%)	With LGA N (%)	χ^2	p
N (%)	30498 (86.2)	4887 (13.8)		
**Maternal age (years)**			329.8	***<.0001***
< 20	7403 (24.3)	777 (15.9)		
20–24	11670 (38.3)	1717 (35.1)		
25–29	6524 (21.4)	1196 (24.5)		
30–34	3497 (11.5)	849 (17.4)		
≥ 35	1404 (4.6)	347 (7.1)		
Mean (SD)	23.8 (5.7)	25.4 (6.0)	-18.6	***<.0001***
**Race**			182.9	***<.0001***
White	15355 (50.4)	2960 (60.6)		
Black	12837 (42.1)	1586 (32.5)		
other	2306 (7.6)	341 (6.9)		
**Parity**			317.3	***<.0001***
0	10115 (33.2)	1051 (21.5)		
1	7255 (23.8)	1147 (23.5)		
≥ 2	13106 (43)	2686 (55.0)		
**SES**			58.4	***<.0001***
1 (Lowest)	2102 (7.1)	269 (5.7)		
2	8832 (29.9)	1250 (26.4)		
3	9096 (30.7)	1460 (30.8)		
4	6173 (20.9)	1134 (23.9)		
5 (Highest)	3384 (11.4)	628 (13.3)		
**Maternal education levels (year)**			11.7	***<.0001***
Less than high school (≤9)	8694 (28.6)	1312 (27.0)		
High school (10–12)	17676 (58.1)	2830 (58.2)		
College and above (>12)	4049 (13.3)	725 (14.9)		
**Married**	23395 (76.7)	4026 (82.4)	77.6	***<.0001***
**Smoking during pregnancy**	14660 (48.5)	1700 (35.1)	299.3	***<.0001***
**Pre-pregnant BMI (kg/m2)**			299.1	***<.0001***
<18.5	4665 (15.6)	538 (11.2)		
18.5–24.9	20021 (66.8)	2935 (61.0)		
>25.0	5299 (17.7)	1340 (27.8)		
Mean (SD)	22.0 (6.8)	23.4 (7.9)	-13.6	***<.0001***
**Gestational weight gain (kg)**			350.4	***<.0001***
< 10.0	14747 (48.7)	1736 (35.7)		
10.0 – 14.9	10896 (36.0)	1980 (40.7)		
≥ 15.0	4660 (15.4)	1151 (23.7)		
Mean (SD)	11.8 (7.9)	13.2 (8.0)	-12.0	***<.0001***
**Pre-existed or gestational diabetes**	424 (1.4)	180 (3.7)	132.3	***<.0001***
**Sex**			0.8	0.400
Male	15391 (50.5)	2499 (51.1)		
Female	15107 (49.5)	2388 (48.9)		

LGA: Large for gestational age.
BMI: body mass index.
SES: socioeconomic status.

Table S7. Risk ratio between adverse outcomes at 7 years of age and pregnant hypertensive disorders, the Collaborative Perinatal Project.

		Without adverse outcome N (%)	With adverse outcome N (%)	Crude OR (95% CI)	Adjusted OR[a] (95% CI)	Adjusted OR[b] (95% CI)
Macrosomia (>4000g)	Normal BP	28699 (85.7)	1610 (83.0)	Ref	Ref	/
	Mild GH + severe GH	3352 (10.1)	236 (12.1)	***1.26 (1.09, 1.45)***	***1.31 (1.12, 1.52)***	
	Mild GH	2968 (8.9)	212 (10.9)	***1.27 (1.10, 1.48)***	***1.32 (1.13, 1.55)***	
	Severe GH	384 (1.2)	24 (1.2)	1.11 (0.74, 1.69)	1.11 (0.69, 1.78)	
	Mild PE	1047 (3.1)	73 (3.8)	1.24 (0.98, 1.58)	1.20 (0.91, 1.57)	
	Severe PE	355 (1.1)	21 (1.1)	1.05 (0.68, 1.64)	1.57 (0.97, 2.52)	
LBW (<2500g)	Normal BP	28827 (85.8)	1482 (81.0)	Ref	Ref	/
	Mild GH + severe GH	3381 (10.1)	207 (11.3)	***1.19 (1.03, 1.38)***	***1.23 (1.04, 1.44)***	
	Mild GH	3009 (9.0)	171 (9.3)	1.11 (0.94, 1.30)	1.16 (0.98, 1.38)	
	Severe GH	372 (1.1)	36 (2.0)	***1.88 (1.33, 2.66)***	***1.71 (1.17, 2.49)***	
	Mild PE	1035 (3.1)	85 (4.7)	***1.60 (1.27, 2.01)***	***1.77 (1.39, 2.25)***	
	Severe PE	322 (1.0)	54 (3.0)	***3.26 (2.43, 4.37)***	***2.77 (2.02, 3.81)***	
LGA (>90th percentile)	Normal BP	26161 (85.8)	4140 (84.8)	Ref	Ref	
	Mild GH + severe GH	3053 (10.0)	535 (10.9)	***1.11 (1.01, 1.22)***	***1.29 (1.16, 1.43)***^	
	Mild GH	2700 (8.9)	480 (9.8)	***1.13 (1.02, 1.25)***	***1.25 (1.12, 1.39)***^	
	Severe GH	353 (1.1)	55 (1.1)	0.99 (0.74, 1.31)	1.08 (0.80, 1.47)^	
	Mild PE	957 (3.1)	163 (3.3)	1.08 (0.91, 1.28)	1.13 (0.94, 1.36)^	
	Severe PE	327 (1.1)	49 (1.0)	0.95 (0.70, 1.28)	1.16 (0.85, 1.60)^	
SGA (<10th percentile)	Normal BP	26773 (86.1)	3528 (82.6)	Ref	Ref	
	Mild GH + severe GH	3111 (10.0)	477 (11.2)	***1.16 (1.05, 1.29)***	1.05 (0.94, 1.18)^	
	Mild GH	2778 (8.9)	402 (9.4)	1.10 (0.98, 1.22)	1.03 (0.91, 1.16)^	
	Severe GH	333 (1.1)	75 (1.8)	***1.71 (1.33, 2.20)***	***1.50 (1.15, 1.95)***^	
	Mild PE	945 (3.0)	175 (4.1)	***1.41 (1.19, 1.66)***	***1.27 (1.07, 1.52)***^	
	Severe PE	287 (0.9)	89 (2.1)	***2.35 (1.85, 2.99)***	***2.00 (1.55, 2.58)***^	
Overweight/obesity at 7 years old	Normal BP	18913 (86.0)	4260 (83.5)	Ref	Ref	Ref

(>85th percentile)	Mild GH	1866 (8.5)	528 (10.3)	***1.26 (1.13, 1.39)***	***1.23 (1.11, 1.37)***	***1.19 (1.07, 1.33)******
	Severe GH	266 (1.2)	63 (1.2)	1.05 (0.80, 1.39)	1.03 (0.77, 1.38)	1.03 (0.77, 1.39)*
	Mild PE	700 (3.2)	190 (3.7)	***1.21 (1.02, 1.42)***	1.16 (0.97, 1.38)	1.15 (0.96, 1.36)*
	Severe PE	251 (1.1)	65 (1.3)	1.15 (0.87, 1.51)	1.10 (0.82, 1.48)	1.13 (0.84, 1.53)*
Elevated SBP at 7 years old	Normal BP	20768 (85.9)	2357 (82.4)	Ref	Ref	Ref
(>90th percentile)	Mild GH	2098 (8.7)	286 (10.0)	***1.20 (1.05, 1.37)***	***1.22 (1.06, 1.39)***	***1.18 (1.02, 1.36)***
	Severe GH	286 (1.2)	43 (1.5)	1.33 (0.99, 1.83)	1.35 (1.00, 1.89)	***1.39 (1.01, 1.96)***
	Mild PE	759 (3.1)	132 (4.6)	***1.53 (1.27, 1.85)***	***1.59 (1.31, 1.94)***	***1.58 (1.29, 1.94)***
	Severe PE	273 (1.1)	43 (1.5)	***1.39 (1.03, 1.92)***	1.31 (0.92, 1.85)	1.32 (0.92, 1.88)
Elevated DBP at 7 years old	Normal BP	20727 (85.7)	2278 (83.2)	Ref	Ref	Ref
(>90th percentile)	Mild GH	2085 (8.6)	293 (10.7)	***1.28 (1.12, 1.46)***	***1.33 (1.17, 1.53)***	***1.29 (1.13, 1.48)***
	Severe GH	286 (1.2)	37 (1.4)	1.18 (0.83, 1.66)	1.33 (0.94, 1.88)	1.30 (0.91, 1.85)
	Mild PE	788 (3.3)	98 (3.6)	1.13 (0.91, 1.40)	1.17 (0.93, 1.46)	1.15 (0.92, 1.44)
	Severe PE	284 (1.2)	30 (1.1)	0.96 (0.66, 1.40)	0.94 (0.62, 1.41)	0.80 (0.59, 1.35)

LBW: Low birth weight.
SGA: Small for gestational age.
LGA: Large for gestational age.
Elevated SBP:Systolic blood pressure at 7 years of age higher than125 mmHg, the 90th percentile within the CPP population.
Elevated DBP: Diastolic blood pressure at 7 years of age higher than 70 mmHg, the 90th percentile within the CPP population.
Overweight/obesity: Race- and sex-specific body mass index at 7 years of age higher than the 85th percentile within the CPP population.
GH: Gestational hypertension.
Mild PE:mild preeclampsia.
Severe PE:Severe preeclampsia/ eclampsia.
Model a: Adjusted for maternal age, parity, race, sex, maternal education level, marital status, smoking status during pregnancy, socioeconomic status, diabetes, maternal pre-pregnancy body mass index (BMI), gestational weight gain, and gestational age.
Model b:Model a + birthweight and body mass index at 7 years of age.
Model ^: maternal age, parity, race, maternal education level, marital status, smoking status during pregnancy, socioeconomic status, diabetes, maternal pre-pregnancy body mass index (BMI), and gestational weight gain.
* Model a + birthweight.
